# Virtual planning for mandible resection and reconstruction

**DOI:** 10.1515/iss-2021-0045

**Published:** 2023-12-06

**Authors:** Florian Andreas Probst, Paris Liokatis, Gerson Mast, Michael Ehrenfeld

**Affiliations:** Department of Oral and Maxillofacial Surgery and Facial Plastic Surgery, University Hospital, LMU, Munich, Germany

**Keywords:** additive manufacturing, CAD/CAM, computer-assisted surgery, craniomaxillofacial surgery

## Abstract

In mandibular reconstruction, computer-assisted procedures, including virtual surgical planning (VSP) and additive manufacturing (AM), have become an integral part of routine clinical practice. Especially complex cases with extensive defects after ablative tumor surgery benefit from a computer-assisted approach. Various CAD/CAM-manufactured tools such as surgical guides (guides for osteotomy, resection and predrilling) support the transition from virtual planning to surgery. Patient-specific implants (PSIs) are of particular value as they facilitate both osteosynthesis and the positioning of bone elements. Computer-based approaches may be associated with higher accuracy, efficiency, and superior patient outcomes. However, certain limitations should be considered, such as additional costs or restricted availability. In the future, automation of the planning process and augmented reality techniques, as well as MRI as a non-ionizing imaging modality, have the potential to further improve the digital workflow.

## Introduction

With respect to mandibular reconstruction, there has been a remarkable shift from conventional procedures to the use of computer-assisted surgery (CAS) over the last ten years, taking advantage of currently available digital technologies. Advances in computing power and improved software applications, as well as advances in 3D printing technology, have contributed to this development. In general, reconstructive mandibular surgery, mainly needed after ablative surgery in an oncological setting, is a challenging task even for the experienced clinician. Successful mandibular reconstruction requires accurate surgical planning and implementation with an appreciation of the underlying complex spatial and functional relationships of the head and neck area [1].

**Table 1:** Guidelines for the evaluation of the accuracy of VSP modified according to van Baar et al. 2019 [34].

Imaging	– Slice thickness <1.25 mm
	– Postoperative scan within 6 weeks and prior to radiation treatment
	– Identical pre-and postoperative machine and scanner parameters
Classification of defects	– Classification of Brown et al. [35]
STL volumes	– Discrepancies of pre-and postoperative STL volumes <0.5 mm
XYZ orientation	– The Frankfurt plane, midsagittal plane, and the nasion should be used
Superimposition of the condylar processes	– The postoperative STL model of the entire reconstruction should be superimposed on the preoperative STL model, with only both condylar processes selected for the iterative closest-point algorithm
Calculation of the angular deviations	– Calculation of the right and left axial, coronal, and sagittal mandibular angles for the pre- and postoperative STL models
Calculation of the *XYZ* deviations of the dental implants	– The correct dental implant diameter and height (including the cover screw) should be used in the preoperative planning for correct comparison. Using the zero reference point on the *XYZ*-axis, the *XYZ*-axis position of the top of the dental implant in the middle of the cover screw is measured with an *XYZ*-deviation plot tool in the pre-and postoperative STL models

In the conventional surgical setting with freehand execution of surgery, planning is based on 2-dimensional information. The surgical implementation itself is of adaptive nature depending on the intraoperative conditions and the use of improvised simple templates. This traditional approach is based mainly on the surgeon’s experience, is time consuming and is prone to inaccuracies. In contrast, computer-assisted surgery implements 3D imaging data as a basis for virtual surgical planning and enables subsequent transfer by CAD/CAM (Computer-Aided Design/Computer-Aided Manufacturing) technologies. This allows for a complete 3-dimensional virtual planning environment.

Virtual surgical planning enables the surgical team to thoroughly study the case and anticipate details and potential problems prior to the operation. Physical 3D printed models can support decision-making and provide options at a very early stage of planning. 3D computer-assisted planning further improves inter-professional exchange as well as communication with the patient. Furthermore, the final prosthetic rehabilitation can be integrated into the digital workflow at an initial stage of reconstructive planning, also called prosthetic-driven backward planning [2].

The aims of mandibular reconstruction are to restore masticatory function, articulation, swallowing and aesthetics. Therefore, restoration of the mandibular and facial shape, including symmetry as well as restoration of intermaxillary relationship and dental rehabilitation, is mandatory [3].

## Indications for mandibular reconstruction

The indications for the reconstruction/construction of the mandible are bone defects, mostly resulting from ablative tumor surgery, but also from infections, trauma or congenital malformations. Segmental defects are the most challenging to address. Different classifications for segmental mandibular defects are used. In a common defect classification of the mandible, according to Urken et al. [4], the mandible is divided into the condylar region (C), ramus (R), mandibular body (B), and symphysis (S). For example, a defect involving the left condyle, ramus, body and the symphysis is represented as S-B-R-C. First and foremost, oral squamous cell carcinomas (OSCCs) infiltrating the mandible are the causative pathology for extensive ablative oncological procedures resulting in segmental mandible defects. Other malignant processes such as osteosarcomas, salivary gland malignancies or distant metastases play a more subordinate role. Benign tumors such as ameloblastoma or other odontogenic tumors and tumor-like lesions can result in segmental defects as well. In addition to neoplastic etiology, osteoradionecrosis and antiresorptive drug-induced osteonecrosis of the jaw (ARONJ) can finally lead to extensive loss of bone substance. Inflammations such as osteomyelitis sometimes also necessitate resections of the mandibular continuity and lead to corresponding defects. Last but not least, complex trauma, such as comminuted fractures, gunshot injuries, or explosion trauma, can cause large mandibular defects. In addition, reconstructive procedures may also be indicated for non-segmental defects such as marginal/rim defects.

## General reconstructive aspects and strategies

Today, microvascular bone transfer is the standard of care for segmental mandibular defects. For basic decision-making, different factors, such as defect localization, defect size and form, mechanical aspects, blood supply of the transplant, possible donor site morbidity, and general medical characteristics of the patient must be taken into account. Depending on the defect configuration and associated characteristics at the recipient site, such as soft tissue conditions, three types of microvascular bone flaps are routinely used [3]. First, the microvascular iliac crest bone flap, which is most suitable for the reconstruction of a dentate mandible, as the vertical dimension can be restored well and thus the prerequisite for the placement of dental implants is favorable. The microvascular fibula flap can be used to bridge particularly long bony mandibular defects as well as combined bone-soft tissue defects. Disadvantages are the limited bone height and the somewhat less reliable vascular supply at the lower leg. Microvascular scapular flaps are characterized by the fact that one or more independently placeable soft tissue islands can also be harvested in addition to the bone portion. The likewise limited bone height and the necessity for intraoperative repositioning may have an unfavorable effect [2].

In order to fixate the bone flap, the application of osteosynthesis plates is common. Load-bearing plates, so-called reconstructions plates, are often used to achieve sufficient stability and secure the position of the remaining mandible elements even if the bony reconstruction fails. In selected cases, these reconstruction plates may also be utilized without bone grafts/flaps for segmental reconstruction. Such bridging of the defect with a reconstruction plate and corresponding soft tissue coverage without bony reconstruction, so-called alloplastic reconstruction, carries a high risk of plate fractures, extraoral and intraoral fistula formation as well as plate exposure in the long term. This concept is therefore not ideal as a permanent solution, especially for irradiated patients. In cases of marginal defects, on the other hand, the application of reconstruction plates alone is often sufficient for adequate biomechanical support [5].

When planning in the mandibular region, it must be considered whether the whole vertical bone dimension needs to be restored. This can most likely be ensured with a microvascular iliac crest flap. However, the maximum achievable length of this flap may be limiting. Furthermore, a stacked fibular flap, the so-called “double-barrel” [6, 7] or “over and under barrel” fibular flap, can provide a good vertical bone height. Alternatively, it may be considered to restore only the crestal bone level, thus ensuring the prerequisite for implant placement with subsequent prosthetic rehabilitation. Restoration of the mandibular basal arch is of secondary functional importance in the posterior region and can take a back seat to crestal reconstruction. On the other hand, in the symphyseal region, esthetic aspects are of greater relevance and attention should be paid to an attractive restoration of the chin contours.

Depending on the bony conditions encountered in the craniomaxillofacial region, the defect configuration, and the dental status, two basic reconstructive strategies can be pursued for the planning sequence: (a) Foreward planning and (b) backward planning.

Foreward planning is the principle of choice in the presence of intact contours and undisplaced, symmetrical contours of the mandible with unchanged jaw relations and non-atrophied dentoalveolar processes. The information derived from the original situation is used as a reference for osteotomies as well as resections and subsequent reconstruction with autogenous bone grafts, bone-containing flaps or allogenic materials. Therefore, “Foreward Planning” or “Prospective Planning” primarily aims to restore the original pre-ablative bone situation, which is significantly altered only after the resection. Foreward planning is, therefore, particularly suitable for primary reconstructions.

In clinical practice, there are often situations in which detailed three-dimensional morphological knowledge of the original skeletal conditions is lacking. In so-called “backward planning” or “reverse planning”, an attempt is then made to use the dimension and arrangement of the presumed original, i.e., regular skeletal architecture, as a template for the subsequent reconstructive procedures. This refers in particular to cases for secondary reconstruction with extensive bony defects or remaining significantly malpositioned fragments. This also applies to patients with congenital malformations in which parts of the facial skeleton may be displaced, malformed or deficient. Accordingly, “backward planning” is particularly suitable for secondary reconstructions.

The term “backward planning” is frequently used specifically for a planning sequence in maxillary or mandibular reconstruction, in which the planning of the optimal positioning of an implant-supported tooth restoration with the setting of a functional occlusion represents the starting point of the entire planning and therefore takes priority. Within the scope of the entire “backward planning” workflow, the positioning of the bone graft/flap components is primarily oriented towards the optimal implant position for a subsequent prosthetic restoration [8, 9].

In such implant-centered/prosthesis-centered/occlusion-centered planning for mandibular reconstruction [10], [11], [12], there are various options for selecting reference points or references to determine the optimal three-dimensional position of jaw segments to be restored. One option is to mirror the alveolar processes of the contralateral mandible or the dental arch with occlusal information. More sophisticated, a virtual tooth setup of the final restoration simulates preliminary implant position based on the position and axes of the digital setup [13]. Subsequently, the position of a bone transplant can be adapted. In conclusion, the “backward planning” concept focuses on implant-supported masticatory rehabilitation, which is the starting point in the entire planning process.

## Digital workflow – overview

**Figure 1:**
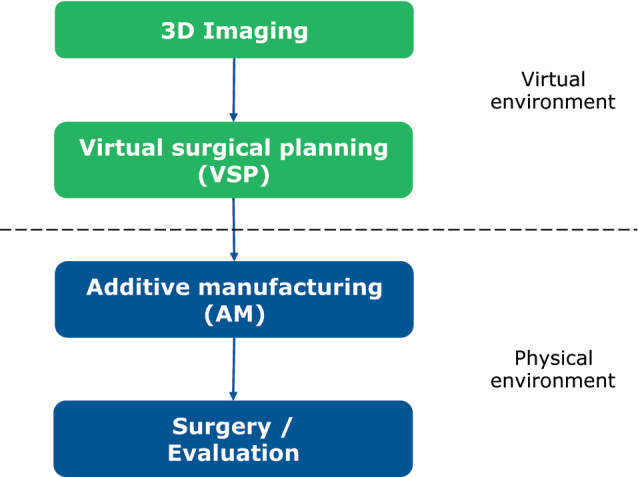
Steps in computer-assisted surgery.

Computer-assisted surgery for mandibular reconstruction is characterized by the following sub-steps: (1) acquisition of 3-dimensional (3D) imaging data, (2) Virtual surgical planning (VSP), (3) Additive manufacturing (AM), and (4) surgical implementation and evaluation (Figure 1). Steps (2) and (3) may also be described by the term CAD/CAM-procedure.

## Imaging and virtual surgical planning

**Figure 2:**
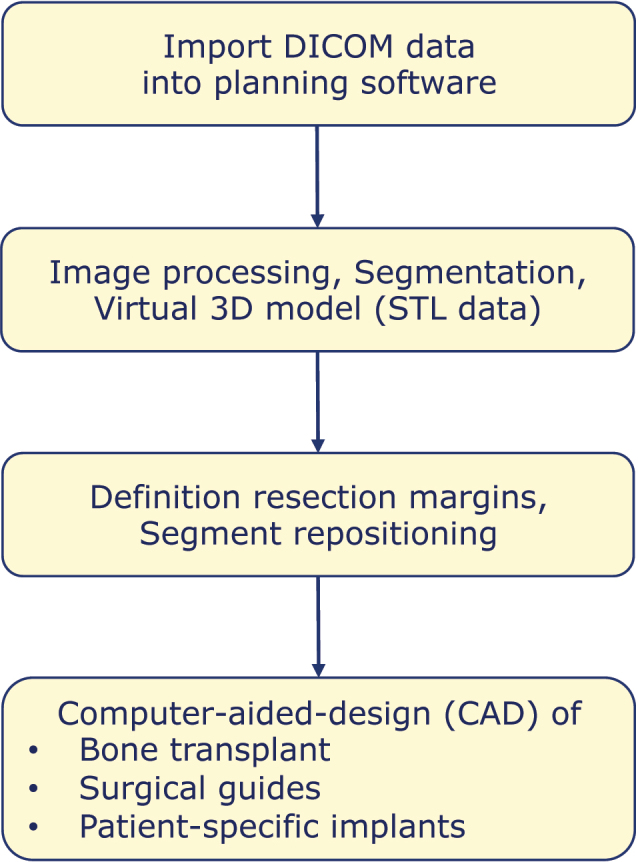
CAD-process in mandibular reconstruction.

The digital workflow of computer-assisted mandibular reconstruction starts with acquiring 3-dimensional, imaging-based information about the patient’s individual morphological situation. Primarily, surgical planning refers to the resection and reconstruction of the mandibular bone conditions and is based on DICOM data sets obtained by computed tomography (CT) or cone-beam computed tomography (CBCT). CT angiography of lower extremities or the scapular region can be obtained as well to simulate the vascular status. High-resolution CT scans with slice increments less than 1.0 mm of the craniomaxillofacial skeleton should be preferred to guarantee proper accuracy [14]. Generic virtual 3D models of the planned donor site may be an acceptable alternative [15]. Imaging-based DICOM (Digital Imaging and Communications in Medicine) datasets are imported into a dedicated 3D planning software followed by image processing (Figure 2). Image segmentation is an essential step in digital image processing, defining anatomical data and pathological information such as a region of tumor infiltration. Finally, DICOM data are converted into virtual 3-dimensional mandibular models and models of the transplant site. 3D models are typically converted into STL data and the resulting files can be converted into physical models by rapid prototyping systems within the CAD/CAM manufacturing process (Figure 2). Additional information may be integrated into the planning process, for example, an optical scan of the occlusion or a virtual dental setup. Superimposing these supplementary data with virtual 3D models of the mandible result in highly detailed so-called “Hybrid models” [13].

In an ablative oncological intervention, resection margins are defined next, taking into account the clinical impression, the multiplanar imaging reconstructions and the required safety margin. The resection can be planned so that the resection surfaces are open caudally and/or laterally, to facilitate insertion of the bone graft. Appropriately beveled resection surfaces can increase the mutual contact surfaces of the graft/flap and the residual mandible elements, which has a beneficial effect on bone consolidation. Likewise, step-shaped resection margins can be designed. Malpositions of the remaining mandibular elements should be reduced accordingly. The definition of resection margins can already be considered part of the CAD process.

CAD includes in particular the virtual reconstruction of the missing parts of the mandible, which is performed in a further step. First, a proper segment is selected in the donor region, keeping a minimum distance from anatomical landmarks such as the lateral malleolus in a fibula graft. In addition, it is decided of how many segments the graft/flap should consist in order to restore the anatomically correct bone contour. Depending on the defect configuration (defect size and localization), the graft/flap is virtually osteotomized, contoured and adjusted to the defect situation. Fibula flaps for reconstruction in the corpus and ramus area usually require an osteotomy to reshape the angle of the mandible. If the condylar region is affected, a condylar head prosthesis can be inserted, attached to a common reconstruction plate systems or as an individual condylar prothesis. Defects that include the symphysis and merge bilaterally into the corpus region often require a three-segment flap. In principle, these defect constellations can be treated with a microvascular fibula, iliac crest or scapular bone flap. Long-range mandibular defects are bridged with bone flaps consisting of up to four or five bone segments, for which fibula flaps are primarily suitable. In parallel to the CAD of the bone tissue, soft tissue aspects such as the planned skin paddle and the localization and length of the recipient vessels must also be considered during planning.

**Figure 3:**
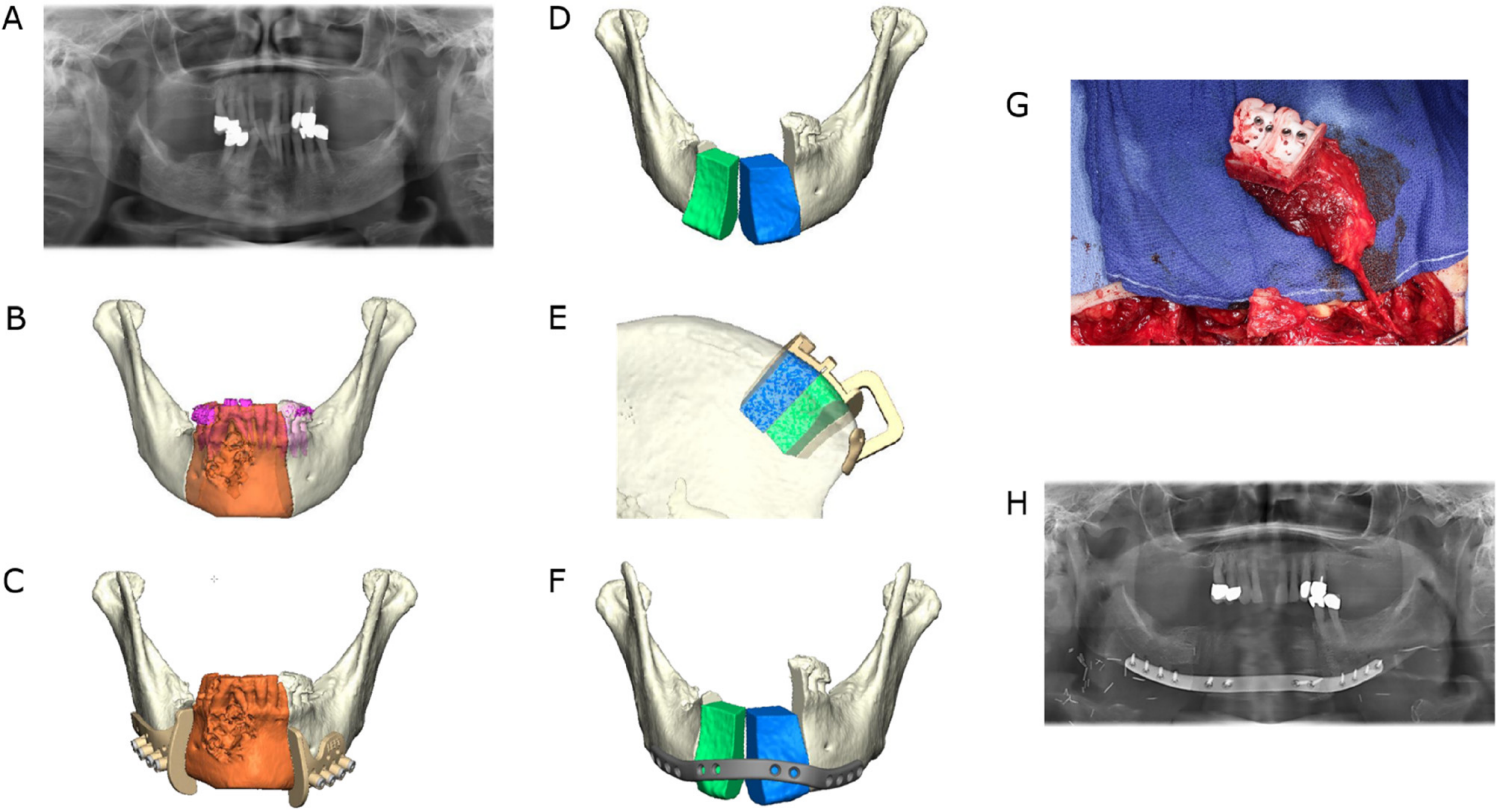
Ameloblastoma of the right symphyseal and body region. Mandibular resection and reconstruction with a microvascular bone flap from the iliac crest is planned. (A) Preoperative panoramic radiograph. (B) Virtual model depiction of the resection area (orange). (C) CAD with combined osteotomy/predrilling guides. (D) Planned reconstruction with ilia crest segments after opening-wedge osteotomy (the gap is later filled with cancellous bone). (E) CAD with osteotomy guide for harvesting the iliac crest transplant. (F) CAD with a patient-specific implant (PSI). (G) Harvested iliac crest flap with osteotomy guide still attached to the vascular pedicle (deep circumflex iliac artery, DCIA). (H) Postoperative panoramic radiograph.

**Figure 4:**
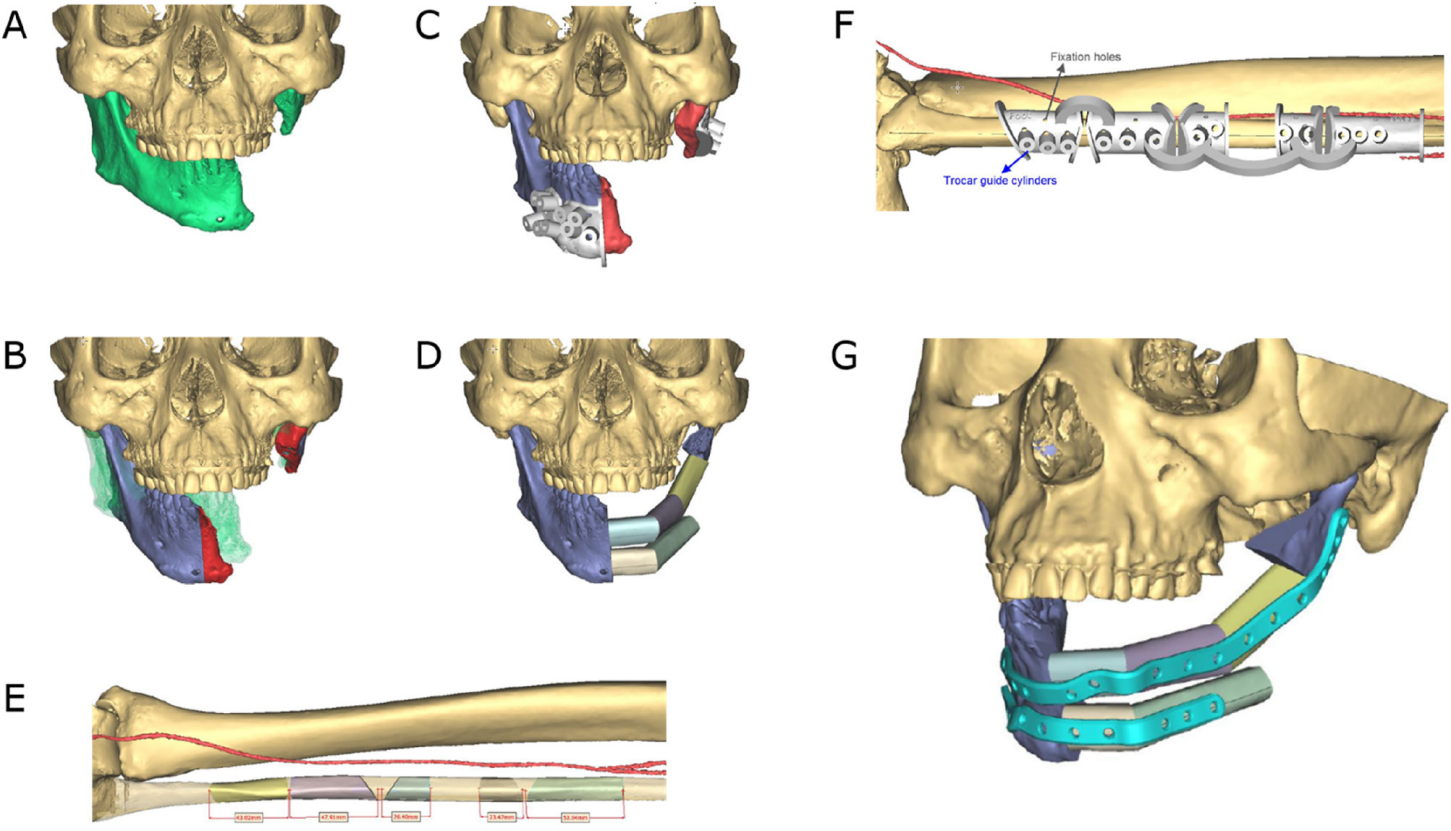
Secondary mandible reconstruction with a double-barrel fibula bone flap and a patient-specific plate (PSI). (A) Virtual 3D model showing the defect area and deviation of the remaining mandible part. (B) Virtual repositioning of the mandible and depiction of the resection area (red). (C) CAD of combined osteotomy/predrilling guides. (D) Planned reconstruction with double-barrel fibula bone flap. (E) Five fibula bone segments are planned. (F) CAD of osteotomy guide for harvesting and segmentation of the fibula bone flap. (F) CAD of a patient-specific implant (PSI).

Moreover, it is important to consider whether the whole vertical bone dimension should be reproduced when planning a mandibular reconstruction. This is most likely to be achieved with a microvascular iliac crest bone flap (Figure 3). However, the maximum achievable length of the bone portion of this flap can have a limiting effect. Furthermore, a layered fibular flap, the so-called “double-barrel” or “over and under barrel” fibular flap, can provide a good vertical bone dimension (Figure 4). Alternatively, only the crestal bone level can be restored according to a prosthetically driven backward planning to ensure an implant placement that enables subsequent prosthetic rehabilitation. The restoration of the mandibular basal arch is of secondary functional importance in the posterior region and can be given secondary importance in favor of crestal reconstruction. In the symphyseal region, on the other hand, aesthetic aspects are of greater relevance and attention should be paid to an attractive restoration of the chin contours.

## Additive manufacturing

The exact transfer of surgical planning from the virtual environment to the surgical situation is a key element in computer-assisted surgery and the prerequisite for clinically satisfying accuracy. This transition from the virtual level to the physical level is enabled by additive manufacturing (CAD/CAM-technology). Navigational systems and intraoperative imaging can be helpful in surgical implementation as well. Different CAD/CAM-manufactured tools supporting the transition process can be employed:

– stereolithography (STL) models
– surgical guides
– patient-specific implants.

**Figure 5:**
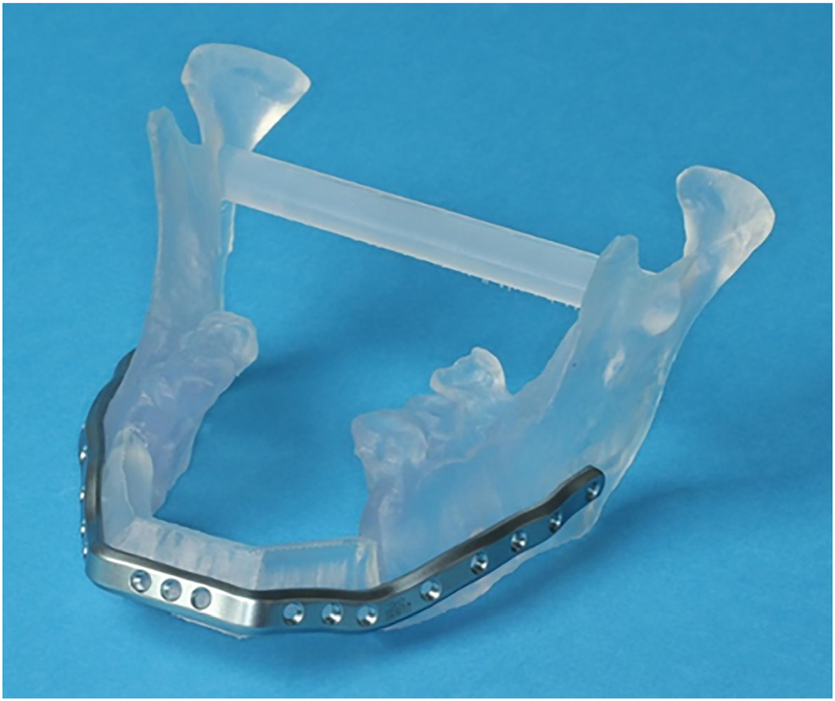
Transparent acrylic STL model including the remaining mandibular elements and the neomandible in the symphyseal area in a case where mandibular resection and reconstruction with a microvascular scapular bone flap is planned.

Stereolithography (STL) models are CAD/CAM-produced replicas of the craniomaxillofacial bone and planned bone transplants (Figure 5). STL models can be used as a template for preoperative manual pre-bending of plates and intraoperative plausibility checks. Moreover, they can be used for illustration for patients as well as for education and training. Common materials for STL models are polyamide and acrylic. Acrylic models can be transparent or dyed in any color to highlight structures such as teeth and nerves.

## Surgical guides

Cutting guides have played an essential role in the transmission of the virtually planned operation since the initial phase of computer-aided surgery.

Following the skeletal rearrangements, the following toolkit of surgical guides may be used:

– repositioning guides and occlusal splints.
– osteotomy (cutting) guides.
– combined predrilling/osteotomy guides for later application of PSI.

Occlusal splints are commonly used in orthognathic surgery but also in mandibular reconstruction. They facilitate the intraoperative repositioning of deviated mandible segments, especially in secondary mandibular reconstruction where jaw segments were malpositioned for a longer period of time. For the same purpose, surgical guides may be implemented that are not fixed to the tooth surfaces but to bony structures of the mandible, so-called repositioning guides.

More important and common is the use of osteotomy guides, also known as cutting guides. Osteotomy guides are usually made of selective laser sintered (SLS) polyamide, but they can also be manufactured from selective laser melted (SLM) titanium. In the context of ablative oncological procedures, these guides are also referred to as resection guides. Segmental resections usually require two osteotomies, one proximal and one distal to the resection area. Osteotomy (resection) guides feature either flanges or slots [16, 17]. Guides can comprise holes used for fixation on the underlying bone with screws during the osteotomy procedure [16, 17] (Figures 3 and 4).

Osteotomy guides (harvesting guides) are also crucial for the harvesting of bone transplants. Several proposals for the design of graft/flap harvesting guides have been made [18], [19], [20]. Standard microvascular bone flaps used for the reconstruction of the mandible are, as already described, the fibula [21], the scapula [16] and the iliac crest graft [22, 23]. After harvesting, the bony parts of the grafts can be segmented and contoured by means of opening or closing osteotomies (Figures 3 and 4).

Care should be taken to enable intraoperative adjustment of resection margins and correspondingly transplant harvesting, if necessary, so that the entire computer-based workflow does not have to be discarded.

## Patient-specific implants (PSI)

Patient-specific implants (PSIs) play a key role in computer-assisted surgery and especially in mandibular reconstruction [17, 24]. They provide stability and contribute to the correct implementation of the reconstruction by enabling a completely digital workflow. They can also be used for alloplastic reconstruction. PSI are typically utilized together with combined predrilling/osteotomy guides. These osteotomy guides come with additionally integrated drilling cylinders with which the screw holes of the later fixed PSI can be predrilled. This technique facilitates the transfer of the PSIs and thus the positioning of the mandibular segments and the transplant position from the virtual planning to the operating theatre. Load-bearing PSIs for mandibular reconstruction are usually made from selective laser melted (SLM) titanium. All in all, PSIs can be regarded to be an effective method for accurate mandibular reconstruction [24, 25]. Compared to conventional reconstruction plates, possible further benefits include high flexibility in plate design and screw placement [26], reduced operating times [25], and potential biomechanical improvements as PSI may be less prone to plate fatigue fractures. Preformed mandibular reconstruction plates are a readily available and cost-effective alternative to PSI [26] (Figures 3 and 4).

## Individual lingual positioned PSI

**Figure 6:**
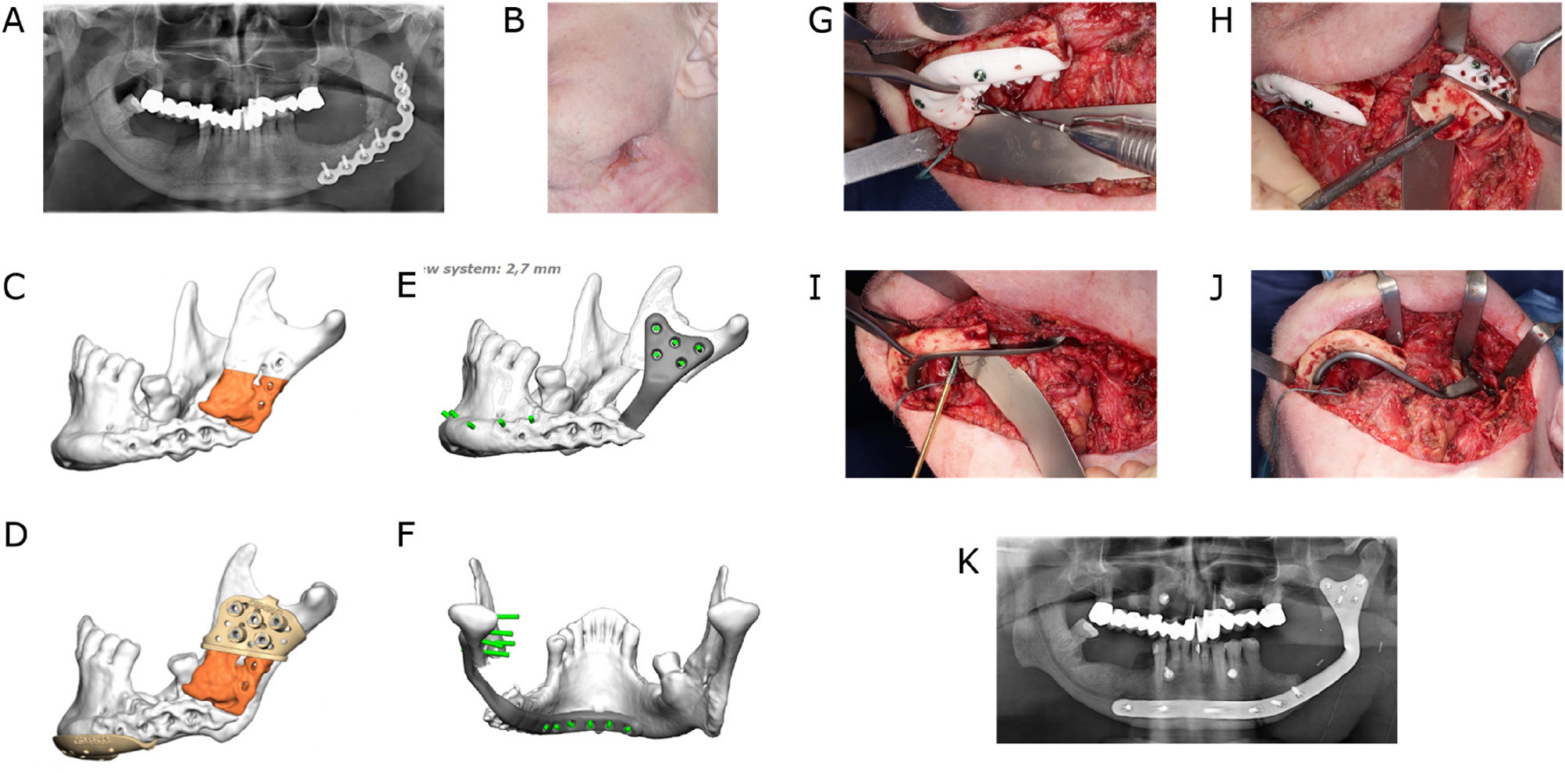
Lingual application of a patient-specific mandible reconstruction plate. (A) Panoramic radiograph of a patient with antiresorptive drug-induced osteonecrosis of the jaw (ARONJ) in the ramus and body region left and a fractured reconstruction plate and (B) compromised soft tissue with cutaneous fistula. (C) Virtual model depiction of the resection area (orange). (D) CAD of combined osteotomy/predrilling guides. (E) and (F) CAD of a patient-specific implant (PSI). (G) Predrilling at the symphyseal area. (H) Resection at the ramus area. (I) Fixation of lingual positioned PSI at the symphyseal area. (J) PSI attached to the mandible. (K) Postoperative panoramic radiograph.

Microvascular reconstruction of a segmental mandibular defect remains the gold standard for functional and aesthetic restoration. However, reconstruction with only a titanium reconstruction plate is necessary in selected cases due to local or systemic limitations. Using reconstruction plates in such a standalone mode to bridge those defects is not a preferable solution, as it may be associated with unpleasant complications such as screws loosening, plate fractures and plate exposure [26]. Virtual surgical planning in conjunction with CAD/CAM-manufactured individualized plates has facilitated the lingual placement of load-bearing osteosynthesis, overcoming many of the aforementioned problems associated with using conventional reconstruction plates [27]. Therefore individualized lingually positioned plates provide an alternative for patients who cannot receive microvascular reconstruction especially in unfavourable soft tissue conditions (Figure 6).

## Dental rehabilitation

**Figure 7:**
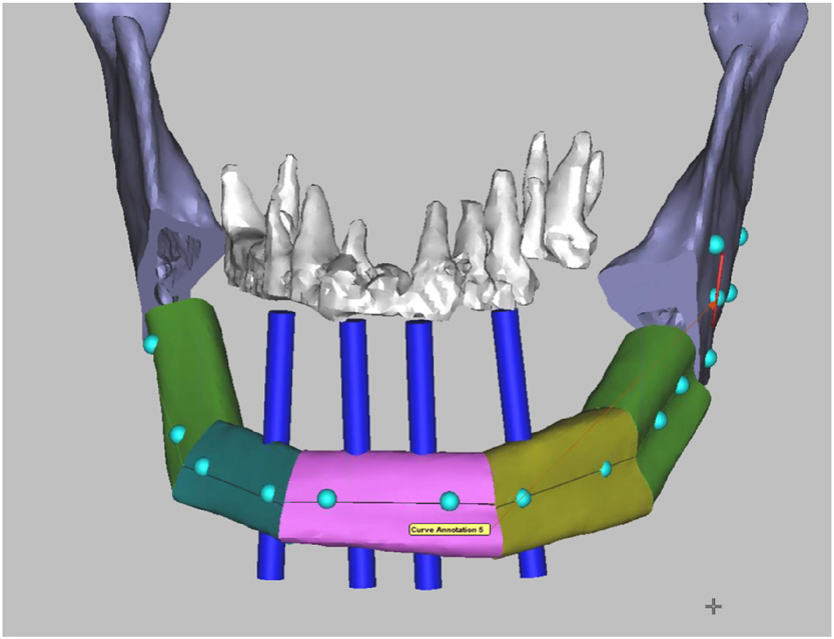
Virtual surgical planning (VSP) for mandibular reconstruction with a fibula bone flap. Four blue dental implant dummies indicate the correct position of the fibula bone segments in relation to the maxillary teeth in order to facilitate the later insertion of dental implants as part of the dental rehabilitation.

The reconstruction of the mandible after ablative surgery aims to restore function and aesthetics. Of crucial importance to these goals is dental rehabilitation. In many studies, it is reported that only a low percentage of patients eventually receive prosthetic rehabilitation. The various reasons include difficulties arising from suboptimal positioning of the grafts/flaps and the challenging new local conditions [28]. With the implementation of 3D planning technology, dental implants can become part of the treatment plan. The bone graft/flap can be placed in a way that facilitates the later placement of implants at the crestal border of the mandibular defect (Figure 7).

Moreover, the VSP allows, together with the improved accuracy and efficacy, the immediate placement of the dental implants or even the prosthetic restoration with the so-called “Jaw in a Day technique” in a single operation [29]. With the use of dental implants in free flaps, issues were raised, including the timing of implant placement in relation to radiotherapy [30]. It has been reported that the placement of implants before radiation therapy is considered a viable option due to an osseointegration time of several weeks before the start of postoperative radiotherapy [31, 32].

## Operation/evaluation

The goals of mandibular reconstruction are to re-establish the aesthetics of the face, restore the patient’s ability to eat, maintain intelligibility of speech, and achieve an accessible airway. These reconstructive goals require a high standard of surgical precision. However, postoperative results never fully match the preoperative virtual plan since inaccuracies are introduced at various stages, including image acquisition, segmentation, 3D printing, surgery, and evaluation of postoperative results [33]. Although individual studies evaluate the accuracy of the VSP, the heterogeneity in planning and evaluation methods raises difficulties for performing valid comparisons between the accuracy results. For this reason, von Baar proposed in 2019 practical guidelines for standardizing evaluation methods to allow comparisons of postoperative outcomes [34] (Table 1).

## Benefits of CAS

Virtual surgical planning (VSP) promises higher accuracy, efficiency, and superior patient outcomes. As mentioned above, comparing the accuracy between different studies is often difficult due to the heterogeneity of the evaluation protocols. A systematic review from 2018, including 413 virtual-planned mandibular reconstructions merely done with free fibula flaps, found a general inaccuracy ranging from 0 to 12.5 mm and 0.9° and 17.5° regarding a plethora of evaluation methods, including distances and angles between various landmarks [33]. However, for the fixation in most of these cases, preoperatively pre-bent or conventional plates were used. Only in 84 patients PSIs were used. This review showed higher accuracy for VSP compared to freehand treatment. Furthermore, VSP plus surgical navigation may produce even greater accuracy.

A meta-analysis from Pucci et al. of relatively homogeneous cases found a mean difference of 2.0 mm for the intercondylar distance and of 3.6° for the gonion angle [36]. Comparatively, in the same meta-analysis, the differences for the freehand reconstructions were 3.9 mm and 7.7°, respectively. Efficiency was assessed by measuring ischemia and total operative time. With data from 121 cases, the mean ischemia time was for VSP 73.8 min, compared with 109.9 min for freehand surgery. The mean total operative time, with 146 total cases, was 291.8 min for VSP, compared with 457.6 min for freehand surgery.

## Drawbacks and limitations of CAS

One of the most significant disadvantages of CAS is the delay caused by preplanning sessions, additive manufacturing as well as shipping of models and guides. With an average time for a planning session of 15–60 min [1, 37] and a delay of 6–18 days [1, 38], the impact on to the treatment due to VSP are less important for most cases of benign tumors and osteoradionecrosis. However, they may have undesired effects on locoregional control of malignant tumors.

Another drawback to VSP, especially for less experienced surgeons, is an over-reliance on cutting guides and patient-specific implants, which can remove flexibility in the operating room, as seen when unexpected intraoperative findings require a change of plans.

Potential sources of error in CAD/CAM-supported surgical procedures exist, for example, in the correct application of osteotomy guides. To avoid errors, anatomical landmarks (e.g. mandibular notch, mental foramen, chin area, jaw angle) and a sufficiently large dimension and support of the guides should be taken into consideration. Furthermore, errors can occur during image segmentation limiting the accuracy of additively manufactured tools such as surgical guides or patient-specific implants (PSI) [39].

Furthermore, in the current literature, there is not too much evidence to which extent the increased accuracy and efficacy from the VSP contributes to an improved clinical outcome for the patient with better functional rehabilitation and aesthetics. Some studies reported that the VSP leads to improved patient satisfaction, but these results should be validated since only in a few studies control groups were examined [21].

The additional costs of digital surgical planning and customized implants is in the mid to high four figures depending on the case’s complexity, and this needs to be weighed against the benefits. Cost analyses on this topic have shown that the benefits can outweigh the costs, especially if a careful case selection is made, using VSP in more complex reconstructions requiring multiple segmentation of bone transplants [40, 41]. For smaller defects, the improved accuracy and efficiency from VSP could be too low to justify the additional costs, especially for an experienced surgeon. However, the costs can be further lowered with solutions such as pre-bending plates on stereolithographic models. It also can be assumed the costs of VSP will continue to decline as the technology is more widely used.

Last but not least, current versions of VSP cannot incorporate the soft tissues into planning and predict the effects to the soft tissue envelope.

## MRI imaging

One major limitation of computer-assisted surgery is that the digital workflow is regularly based on CT and CBCT, which expose patients to ionizing radiation. Magnetic resonance imaging is an alternative 3D imaging technique without radiation exposure. Advantages in MRI technology resulted in significantly improved imaging of hard tissue such as bone and teeth, and therefore, MRI is rapidly gaining momentum in craniomaxillofacial surgery and dentistry [13, 42], [43], [44], [45], [46]. MRI is of particular interest as an imaging basis for computer-assisted surgery and has proven his principal suitability to create accurate virtual 3-dimensional bone surface models of the mandible [39, 47]. Furthermore, MRI can add value over CT because of its superior soft-tissue contrast. Structures such as mucosa, neurovascular structures, skin and subcutaneous tissue may be better implemented in the virtual planning process [48, 49]. Last but not least also pathologically altered tissue with inflammation can be visualized [50], and there may be a benefit for depicting tumor invasion of the mandibular bone.

## Artificial intelligence

Virtual surgical planning is becoming increasingly important in surgical treatment and it can be assumed that the procedure will continue to evolve. Until now, commercially available software does not offer any additional information about essential considerations of a reconstruction [51]. Matters such as which transplant is best suited for a specific defect, the number of osteotomies needed, the length of the segments, the preferable placement of the bony part of the transplant, the position of soft tissues or the vascular pedicle are often addressed on an online-meeting between a surgeon and a software engineer and are mostly left on the knowledge and experience of the surgeon to decide. The introduction of artificial intelligence with appropriate algorithms to make suggestions on these crucial parameters has great potential to automatise VSP, significantly reduce the time and effort required and improve outcomes. Modabber et al. suggested and evaluated an algorithm for automating the VSP of fibula transfer, showing that this technology is applicable and can simplify the procedure [52, 53]. Automating digital planning will probably further decrease the costs of VSP. Moreover, it will simplify the technology, and through it, the surgical procedure of a microvascular reconstruction of the mandible may find its way into the daily routine of centers and surgeons that do not yet exercise it.

## Virtual reality (VR) and augmented reality (AR)

Virtual reality (VR) is a simulated experience that facilitates the visualization of digital information in three dimensions [54]. VR-applications can contribute to the planning of maxillofacial procedures and surgery training [55], [56], [57].

Augmented reality, in contrast to VR, incorporates the real-world environment into the experience of the user. In other words, while VR moves the user to a virtual environment, AR moves digital information on objects or places in the real world in real-time [58]. AR is a promising technique to transfer virtual surgical planning into the operation as an alternative to established navigational systems. There are two ways to transfer the digitally planned surgery to the operation theatre and the patient and possibly improve the achieved accuracy [56]. It can be used either for positioning the surgical guides and thus the transplant or for direct positioning the transplant itself. The positioning of the guides or the segments can be achieved by two techniques: either by the pairing of assigned reference points or by matching the surface contours [59]. Feasibility studies show how augmented reality technology can support mandibular resection and fibula flap harvesting [60, 61]. Nevertheless, AR technology still needs to be significantly improved before it can be used in clinical routine.

## Conclusions

Computer-assisted mandibular reconstruction, including virtual surgical planning and additive manufacturing, is well established, particularly for free flap reconstruction. Especially complex cases with large defects after ablative tumor surgery benefit from a computer-assisted approach. Various CAD/CAM-manufactured tools such as surgical guides (guides for osteotomy, resection and predrilling) effectively support the transition process from virtual planning to surgery. Additionally, patient-specific implants (PSIs) can be used to enable a completely digital workflow and facilitate both osteosynthesis and the positioning of bone elements. Computer-assisted mandibular reconstruction is reported to result in higher accuracy and efficiency and superior clinical outcomes. However, certain limitations should be considered, such as additional cost, restricted availability and delay of surgery. Automation of virtual planning, implementation of augmented reality techniques, and MRI as an imaging alternative may contribute to further improvements.

## Supplementary Material

Supplementary Material
