# Supplementary material for: Virtual planning for mandible resection and reconstruction
Source: Innov Surg Sci. 2023 Dec 6;8(3):137–48. doi: 10.1515/iss-2021-0045 (PMC10709695; doi:10.1515/iss-2021-0045)
Supplement: Supplementary file 1 [file j_iss-2021-0045_suppl_001.pdf]

Reviewer Assessment

J. Pfannschmidt et al.: Extracapsular extension of pN2 lymph node metastases is not prognostically significant in surgically resected patients with non-small cell lung cancer

Reviewers' Comments to Original Submission

Reviewer 1: anonymous

Date received: 13-Jan-2023
Reviewer recommendation: **Return to author for minor modifications**
Reviewer overall scoring: **High**

Comments to author:
The Dear authors, thank you for submitting this paper.

I just have a few minor points which needs to be cleared:

How often did you find a fragmented lymph node tissue? Percentage of all Non-ENE?
What operative access is used (open, VATS, RATS?)
Can you please discuss possible consequences of more modern diagnostic tools (liquid biopsy) and immunotherapy chances

Reviewer 2: Kugler, Christian

Date received: 28-Feb-2023
Reviewer recommendation: **Accept in present form**
Reviewer overall scoring: **Medium**

Comments to author:
General:
The study cohort is relatively small and the total number of patients is probably too small to be a prognostic factor.
There is no universal definition of ENE, even for routine pathological examination of lymphatic compartments there are no international standards. This makes comparability with other studies almost impossible.

Authors' Response to Reviewer Comments

Date received: 03-Mar-2023

Response to reviewer 1:

- The reviewer asked how often we found fragmented lymph node tissue.

In 78 of 81 ENE positive lymph nodes, the lymph node was complete. In three cases the lymph node was fragmented, but after careful examination by the pathologists it was possible to reconstruct enough to determine that the affected fragments belonged to single lymph node so that they were not erroneously counted twice.

However, we do not have the exact number of fragmented lymph nodes for all lymph nodes dissected available. 67 patients (57%) were negative for ENE. Here, we counted a total of 157 lymph node specimens; 24 of these were lymph-node fragments (15%), and the rest were complete lymph nodes. We have added this information to our results section.

- The reviewer asked about the percentage of all Non-ENE.

Of the 1800 lymph nodes examined, 81 were ENE+. Thus, in 95.5% no ENE was detected. This information was added to the manuscript. We have added this information to our results section.

- The reviewer asked about the surgical procedures used (open thoracotomy, VATS, RATS?).

In our study 115 procedures were performed via lateral open thoracotomy and three were minimally-invasive (VATS). There were no RATS dissections. We have added this information to our manuscript.

As suggested by the reviewer we have added a brief discussion of the consequences of more modern diagnostic tools (liquid biopsy) and immunotherapy..

Response to reviewer 2:

- The reviewer suggested that the study cohort is relatively too small for examining prognostic factors.

We agree that the number of patients is too small for any decisive conclusions . However, as we stated in the limitations section (at the end of our manuscript) our hope is to inspire interest in considering ENE-status as part of the decision making-procedure for adjuvant therapies (i.e. PORT) in the postoperative multidisciplinary tumor board.

- The reviewer asked about the definition of ENE, because there is no universal definition of ENE, even for routine pathological examination of lymphatic compartments.

We acknowledge the dilemma that there is no clear definition of ENE, which limits comparison of ENE between different publications. We have mentioned this dilemma in our discussion, as well as our rational for the definition that we used. The separate definition for ENE as metastatic cells appearing to extend continuously beyond the lymph node capsule or where it was defined by including tumor deposits and vascular emboli observed in the surrounding adipose tissue was fully addressed in the discussion section. We have highlighted this problem in the discussion and the need for a consensus definition for future studies.

Reviewers’ Comments to Revised Submission

Reviewer 1: anonymous

Date received: 13-Mar-2023

Reviewer recommendation:	**Accept in present form**
Reviewer overall scoring:	**High**

Comments to author:

Thank you for the revised and corrected Paper. No further comments

Reviewer 2: Kugler, Christian

Date received: 16-Mar-2023

Reviewer recommendation:	**Accept in present form**
Reviewer overall scoring:	**Medium**

Comments to author:

I in the revised form, I now plead for the publication
